# Diseases Admitted under the Care of the Physicians of the Westminster Hospital, from the 24th of September to the 22d of October, 1800

**Published:** 1800-11

**Authors:** 


*Diſeaſes admitted under the Care of the Phyſicians of the Weſtminſter Hoſpital, from the 24th of September to the 22d of October,* 1800.

| | | | |
|---|---|---|---|
| Continued Fever | 15 | Aſcites | 2 |
| Intermittent | 1 | Aſthenia | 3 |
| Pleuriſy | 1 | Catarrh | 1 |
| Amenorrhœa | 3 | Cephalæa | |
| Anaſarca | 3 | Colic | 1 |

Cough

| | | | |
|---|---|---|---|
| Cough | 9 | Lumbago | 1 |
| Diarrhœa | 12 | Menorrhagia | 1 |
| Dyfpepfia | 2 | Phthifis | 3 |
| Dyfpnœa | 1 | Pleurodynia | 1 |
| Dyfuria | 2 | Pleurigo | 1 |
| Enterodynia | 1 | Rheumatifm | 7 |
| Epilepfy | 1 | Struma | 2 |
| Hæmatemefis | 1 | Vomiting | 2 |
| Lepra Græc. | 2 | Worms | 1 |
| Leucorrhœa | 1 | | |

